# Arbuscular Mycorrhiza Fungi Reduce Photosystem II Efficiency in Phosphorus-Deficient Maize Without Promoting Growth

**DOI:** 10.3390/plants15142215

**Published:** 2026-07-21

**Authors:** Luqman Dau, Arunee Wongkaew, Wannasiri Wannarat, Apidet Rakpenthai, Worachart Wisawapipat, Orawan Kumdee, Sirilak Kaewsuralikhit, Sutkhet Nakasathien

**Affiliations:** 1 Department of Agronomy, Faculty of Agriculture, Kasetsart University, 50 Ngamwongwan Road, Lat Yao, Chatuchak, Bangkok 10900, Thailand; luqmanramadhani.d@ku.th (L.D.); fagranw@ku.ac.th (A.W.); wannasiri.w@ku.th (W.W.); fagradr@ku.ac.th (A.R.); 2 Department of Soil Science, Faculty of Agriculture, Kasetsart University, 50 Ngamwongwan Road, Lat Yao, Chatuchak, Bangkok 10900, Thailand; worachart.w@ku.ac.th; 3 Agricultural Research and Technology Transfer Center, Faculty of Agriculture, Kasetsart University, Bangkok 10900, Thailand; fagrowk@ku.ac.th; 4 Soil Microbiology Research Group, Soil Science Group, Agricultural Production Sciences Research and Development Division, Department of Agriculture, 50 Phahonyothin Road, Lat Yao, Chatuchak, Bangkok 10900, Thailand; sirilak@doa.in.th

**Keywords:** arbuscular mycorrhiza fungi, phosphorus deficiency, relative growth rate, net assimilation rate, leaf area ratio, photosystem II

## Abstract

Arbuscular mycorrhizal fungi (AMF) are widely reported to restore photosynthetic efficiency under phosphorus deficiency. Whether this holds under combined phosphorus-and-zinc deficiency in early-stage maize (*Zea mays*), and how AMF modify the relationship between photosynthetic efficiency and growth rate, remains unresolved. Maize variety SUWAN 5819 was grown under five nutrient treatments (+Zn+P, −Zn−P, +Zn−P, −Zn+P, deionized water) with and without AMF in a sand culture under a randomized complete block split-plot design with six replications. Chlorophyll fluorescence, stomatal conductance, and growth-analysis variables were measured. Lindeman–Merenda–Gold (LMG) variance partitioning and piecewise structural equation modelling (SEM) decomposed the relative growth rate (RGR) into net assimilation rate (NAR) and leaf area ratio (LAR) components. AMF reduced the quantum efficiency of photosystem II (ΦPSII) by 29.6–30.8% in phosphorus-deficient treatments. Despite this, AMF had no significant effect on NAR, LAR, or RGR across any treatment. NAR contributed 62.9–99.2% of explained RGR variance across all treatment combinations, consistently exceeding LAR. Under combined −Zn−P deficiency, stomatal conductance was significantly elevated despite reduced ΦPSII and ETR. The piecewise SEM showed that AMF caused a 1.7 times increase in the negative effect of −Zn−P on ΦPSII. This study shows that phosphorus is the main determinant of the quantum efficiency of photosystem II whilst AMF under phosphorus deficiency reduce photochemical efficiency without a compensating growth benefit in early vegetative maize.

## 1. Introduction

Maize (*Zea mays* L.) is one of the most important cereal crops globally, grown on over 200 million hectares with a production output exceeding 1.2 billion tons as of 2024 [1,2,3]. Despite this importance, maize production is severely constrained by abiotic stresses. Among the leading constraining factors is the bioavailability of zinc (Zn) and phosphorus (P) in agricultural soils [4,5,6,7]. A substantial component of this limitation operates through impaired photosynthetic performance, as both nutrients are directly involved in the processes that determine carbon assimilation [8].

Zinc deficiency has adverse effects on the photochemical components of photosynthesis. The deficiency has been previously reported to reduce photosystem II activity by 37% in maize relative to a control [9], a finding supported by [10], who observed a comparable 23.1% reduction. A study on early vegetative maize revealed a decrease in all physiological parameters under zinc starvation, with physiological symptoms appearing before anatomical ones [11]. A study on dual deficiency of iron and zinc [12] found that iron deficiency and combined iron-and-zinc deficiency significantly reduced the quantum efficiency of PSII (ΦPSII) relative to the +Fe+Zn control at 12 days after transplanting, with no significant difference between the two deficiency treatments. The study further noted that the results in the absence of zinc alone, however, did not differ significantly from the control. A study on water stress combined with zinc stress in early maize showed lower PSII quantum efficiency under the combined stress [13]. Mechanistically, zinc stress reduces photosynthetic rate through overproduction of reactive oxygen species (ROS), which damage the light-harvesting complex, impair PSII reaction centers, and cause chloroplast membrane degradation [14].

Phosphorus deficiency similarly reduces the quantum yield of PSII [15,16,17]. A study conducted on early vegetative maize reported as much as an 86% reduction in PSII quantum efficiency during phosphorus starvation [18]. Low inorganic phosphate (Pi) concentration in the stroma reduces ATP synthase activity, leading to thylakoid lumen acidification and impaired electron transfer, with the consequence that ΦPSII and electron transport rate (ETR) are both reduced [19,20]. This is further compounded by reduced availability of ATP and NADP^+^ directly impairing the photochemical reactions of photosynthesis [19,21]. Zinc and phosphorus deficiency therefore converge on reductions in ΦPSII and ETR through separate mechanisms, with downstream consequences for plant growth.

Both zinc and phosphorus stress have been reported to reduce stomatal conductance, with findings being less in agreement. A study by [22] reported a decrease in stomatal conductance in zinc starvation consistent with an earlier study by [13]. However, contrasting results were reported by [12], noting no significant difference in stomatal conductance between −Zn and the control for early maize. Phosphorus deficiency reduced stomatal conductance in early maize [15] and was also observed to reduce stomatal conductance in cotton [23]. A study on different rates of phosphorus in maize at the vegetative stage showed an increase in stomatal conductance with an increase in phosphorus supply for five maize hybrids [24]. Unlike in most studies, the stomatal conductance between low phosphorus treatment and the control (sufficient phosphorus) was comparable in early vegetative maize in a study by [18].

Arbuscular mycorrhizal fungi (AMF) are obligate biotrophs that develop an extensive extraradical hyphal network from which they enhance host-plant uptake of immobile nutrients including phosphorus and zinc [25,26]. AMF develop hyphae beyond the rhizosphere, and due to their small diameter, can access small soil pores that are inaccessible to the roots [27]. AMF have been recorded to improve growth and development, including photosynthetic parameters. Studies by [28,29,30,31] with different levels of phosphorus and zinc showed a higher stomatal conductance with AMF maize compared to non-AMF maize.

Quantifying the physiological consequences of nutrient deficiency on plant growth requires a framework that attributes differences in biomass accumulation to their underlying determinants. The relative growth rate (RGR) analysis framework provides such a decomposition [32]. RGR is defined as the increase in biomass per unit of existing biomass per unit time and can be resolved into two primary components, RGR = NAR × LAR, where the net assimilation rate (NAR) represents the net rate of dry matter production per unit leaf area, and the leaf area ratio (LAR) represents the ratio of photosynthetically active leaf area to total plant biomass. RGR, LAR, and NAR have been used as growth indices to show the effects of abiotic stress in plants [33,34,35,36]. However, the determination of the contribution of NAR and LAR to RGR has not been explored in zinc and phosphorus deficiencies or the combination of the two in the presence of mycorrhiza. We hypothesized the following in this study: (i) AMF will improve the quantum efficiency of photosystem II and ETR for nutrient-starved treatments; and (ii) NAR’s contribution to RGR will be greater than that of LAR in nutrient-deficient treatments with and without AMF.

The objectives of this study were: (i) to quantify the effects of zinc and phosphorus deficiency, with and without AMF inoculation, on stomatal conductance, ΦPSII, and ETR in maize during the early vegetative stage; and (ii) to determine the relative contribution of NAR and LAR to RGR under these treatment combinations in early vegetative maize.

## 2. Results

### 2.1. AMF Root Colonization

AMF inoculation significantly increased root colonization across all nutrient treatments relative to uninoculated controls (*p* < 0.001; Table 1), with colonization in M1 treatments being 5–12 times higher compared to M0. Neither the nutrient treatment effect nor the nutrient × AMF interaction was statistically significant, indicating that colonization levels did not differ among nutrient treatments within inoculated plots. Although the M0 treatments had detectable colonization (<5.7%), the large difference (5–12 times) demonstrates that the residual colonization did not affect data interpretation.

### 2.2. Stomatal Conductance, ΦPSII, and Electron Transport Rate

**Figure 1:**
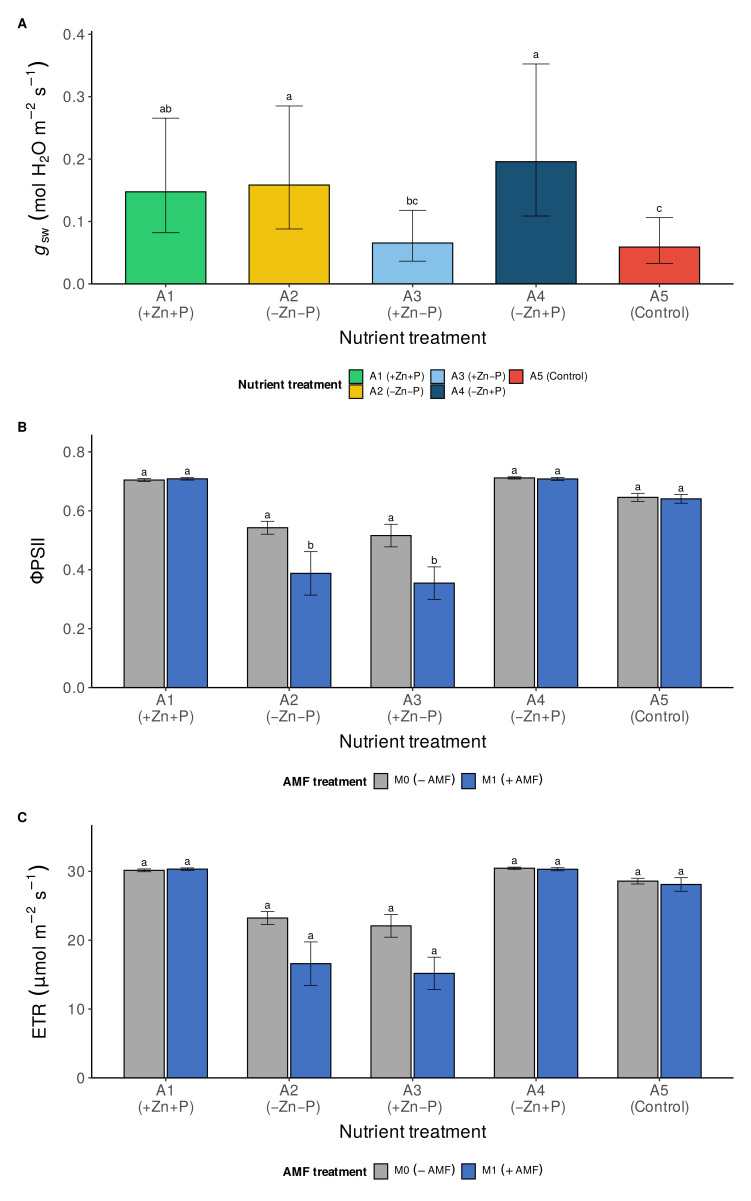
Effects of zinc and phosphorus nutrient treatments on (**A**) stomatal conductance, (**B**) ΦPSII with and without AMF inoculation, and (**C**) electron transport rate (ETR) with and without AMF inoculation. Compact letter displays indicate significant pairwise differences by Šidák adjustment at *p* < 0.05 where bars sharing a letter are not significantly different. M0 = without AMF; M1 = with AMF.

Nutrient treatment significantly affected stomatal conductance, ΦPSII, and ETR (Table 2; Figure 1). The phosphorus-sufficient treatments (A1 and A4) showed the highest ΦPSII and ETR values, with AMF having no significant effect under these conditions. AMF inoculation significantly reduced both ΦPSII (*p* < 0.01) and ETR (*p* < 0.001) in the A2 (29.6% and 28.4%, respectively) and A3 (30.8% and 31.2%, respectively) treatments, and a significant nutrient × AMF interaction was found for both variables. The −Zn−P treatment (A2) showed anomalously high stomatal conductance relative to its ΦPSII and ETR values. There was no significant effect of AMF on stomatal conductance.

### 2.3. Dry Matter Production

Nutrient treatment significantly affected all dry weight parameters at both harvests: harvest 1 at 15 days after sowing (DAS) and harvest 2 at 22 days after sowing (DAS) (*p* < 0.001; Table 3). The phosphorus-sufficient treatments (A1 and A4) consistently produced the greatest leaf, stem, root, and total dry weights at both sampling dates, whereas the phosphorus-deficient treatments (A2, A3) and the deionized water control (A5) produced lower dry weights. AMF inoculation significantly affected root dry weight (*p* < 0.01) and total dry weight (*p* < 0.001) at harvest 2, and a significant nutrient × AMF interaction was found for all dry weight components at this harvest. These effects were specific to absolute biomass at the second harvest and were not detected at harvest 1, where AMF had no significant effect on any dry weight component.

### 2.4. Leaf Area, Growth Rates, and Net Assimilation Rate

Nutrient treatment significantly affected leaf area, LAR, all organ-level RGRs, whole-plant RGR, and NAR (*p* < 0.001; Table 4). AMF inoculation had a significant effect on LAR (*p* < 0.05), but no significant nutrient × AMF interaction was found for any of the growth variables despite significant nutrient × AMF interaction at the second harvest for root and total dry weights. Thus, the AMF-related differences in root and total dry weights at the second harvest were not accompanied by corresponding differences in the estimated RGR.

**Figure 2:**
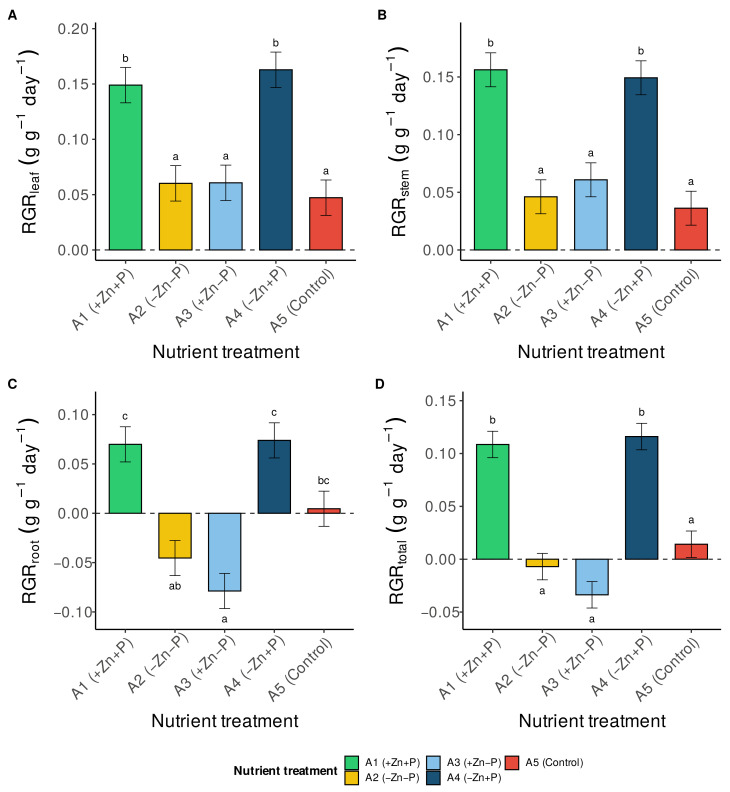
Relative growth rates for (**A**) leaf, (**B**) stem, (**C**) root, and (**D**) whole plant, calculated from dry weights measured at two destructive samplings seven days apart. Compact letter displays indicate significant pairwise differences by Šidák adjustment (*p* < 0.05) where bars sharing a letter are not significantly different. Treatment codes: A1 = +Zn+P; A2 = −Zn−P; A3 = +Zn−P; A4 = −Zn+P; A5 = deionized water control.

**Figure 3:**
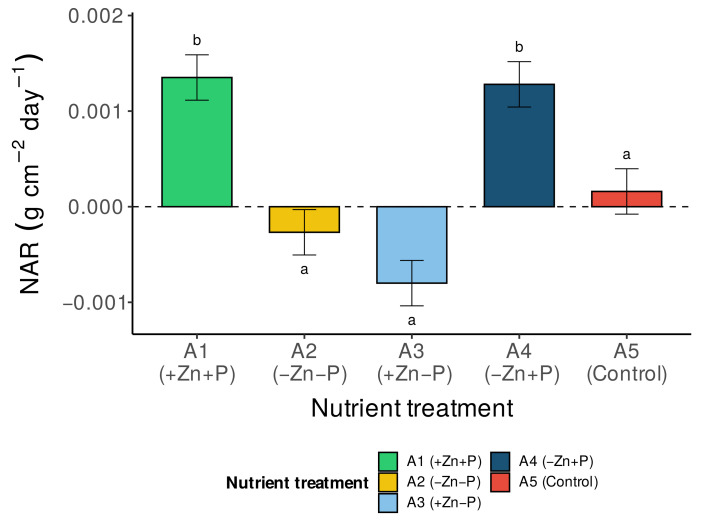
Net assimilation rate (NAR) for each nutrient treatment, calculated from destructive samplings seven days apart. Compact letter displays indicate significant pairwise differences by Šidák adjustment (*p* < 0.05) where bars sharing a letter are not significantly different. Treatment codes: A1 = +Zn+P; A2 = −Zn−P; A3 = +Zn−P; A4 = −Zn+P; A5 = deionized water control.

The phosphorus-sufficient treatments (A1, A4) showed positive RGRs and NAR values. Conversely, the phosphorus-deficient treatments (A2, A3) showed near-zero or negative root and whole-plant RGRs over the seven-day inter-harvest period. Consistent with the declining RGRs in the phosphorus-deficient treatments was the negative NAR, consistent with declining photosynthetic efficiency. Whole-plant RGR most closely followed the pattern of root RGR (Figure 2 and Figure 3).

### 2.5. Principal Component Analysis

**Figure 4:**
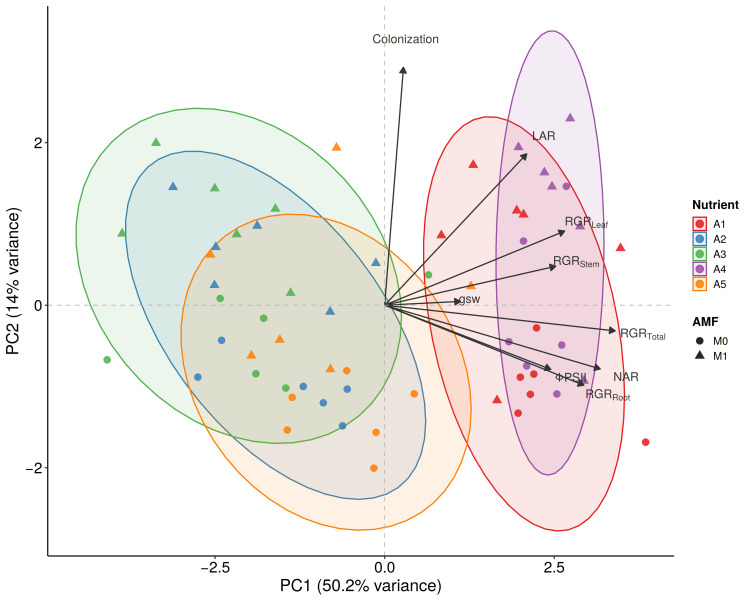
Principal component analysis (PCA) biplot of nine maize physiological and growth parameters showing principal component 1 (PC1) (50.2%) against principal component 2 (PC2) (14.0%). Circles = without AMF (M0); triangles = with AMF (M1). Ellipses represent 95% confidence regions for each treatment combination. LAR = leaf area ratio; RGR = relative growth rate; gsw = stomatal conductance; NAR = net assimilation rate; ΦPSII = effective quantum yield of photosystem II. A1 = +Zn+P; A2 = −Zn−P; A3 = +Zn−P; A4 = −Zn+P; A5 = deionized water control.

Together, principal component 1 (PC1) and principal component 2 (PC2) explained 64.2% of the variance among the nine variables examined in colonization, growth (leaf, stem, root, and whole-plant RGRs, NAR, LAR), and physiology (stomatal conductance, ΦPSII) (Figure 4). PC1 (50.2%) separated treatments along a growth axis, with phosphorus-sufficient treatments clustering to the right, characterized by positive RGR and high values of NAR and ΦPSII, and phosphorus-deficient treatments clustering to the left irrespective of AMF. The acute angle between the NAR and whole-plant RGR vectors indicated a stronger association between these two variables as compared to LAR and whole-plant RGR. This observed pattern has been quantified in Section 2.6. Moreover, the proximity of root RGR to the whole-plant RGR vector indicated that root growth rate was the primary driver of whole-plant growth dynamics. The short colonization vector indicated limited overall influence of inoculation on multivariate growth patterns. PC2 (14.0%) was defined primarily by leaf morphology (stomatal conductance and LAR). The acute angle between AMF colonization and LAR suggests a greater association of AMF with leaf morphology than with stomatal physiology.

### 2.6. Relative Importance of NAR and LAR

The closer alignment of NAR with RGR relative to LAR observed in the PCA (Section 2.5) was quantified using Lindeman Merenda Gold (LMG) variance partitioning. The analysis identified NAR as the dominant contributor to variation in whole-plant RGR across all nutrient and AMF treatment combinations (Table 5). Contributions ranged from 62.9% (A4M1) to 99.2% (A5M1) for NAR, compared with 0.8% (A5M1) to 37.1% (A4M1) for LAR. LAR’s relative contribution was greatest in the A4 (−Zn+P) treatment with AMF inoculation.

### 2.7. Piecewise Structural Equation Model (SEM)

**Figure 5:**
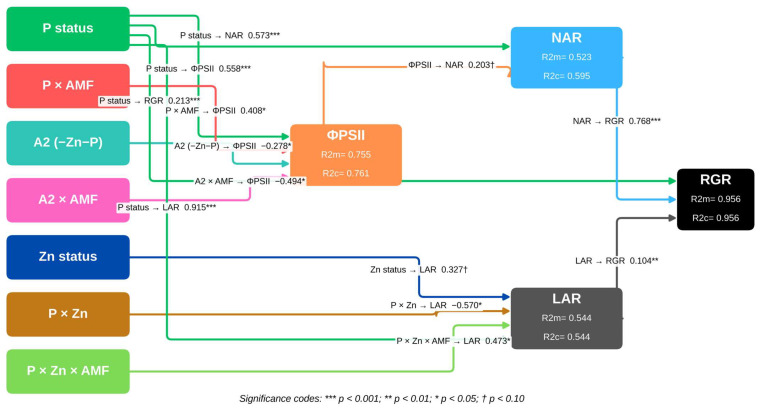
Piecewise structural equation model (SEM) path diagram showing the direct and indirect effects of P supply status, Zn supply status, and AMF inoculation on whole-plant relative growth rate (RGR) mediated through ΦPSII, net assimilation rate (NAR), and leaf area ratio (LAR). Arrow widths are proportional to the magnitude of standardized path coefficients (β). Significance codes: *** *p* < 0.001; ** *p* < 0.01; * *p* < 0.05; † *p* < 0.1.

The piecewise SEM was used to map the causal structure of NAR and LAR contributions to RGR and to test for pathways not captured by the NAR/LAR decomposition. The model revealed three significant pathways through which phosphorus availability influenced whole-plant RGR (Table 6; Figure 5). The first and dominant pathway was via P status → NAR (β = 0.573) → RGR (β = 0.768). The second pathway was via P status → LAR (β = 0.915) → RGR (β = 0.104). The third was a direct pathway from P status to RGR (β = 0.213) independent of NAR and LAR. Phosphorus was 1.3 times less effective on ΦPSII with the presence of AMF (β = 0.558 → 0.408). Dual deficiency had a negative impact on PSII (β = −0.278), with the effect increasing 1.7 times with the dual deficiency–AMF interaction (β = −0.494). Phosphorus interaction with zinc had a negative impact on LAR (β = −0.570), with phosphorus–zinc–AMF interaction resulting in a positive influence on LAR β = 0.473). Zinc status had no significant direct effect on ΦPSII in the model.

## 3. Discussion

### 3.1. Phosphorus Deficiency Is the Primary Determinant of ΦPSII and ETR

Phosphorus deficiency, not zinc deficiency, drove the photochemical impairment recorded at 22 days after sowing. Phosphorus-deficient treatments showed reduced ΦPSII and ETR, while zinc status alone produced no comparable effect. Reduced photochemical activity has been shown to result in low stromal Pi that suppresses ATP synthase activity and acidifies the thylakoid lumen, thereby impairing electron transfer [19,21,37,38,39,40]. Moreover, low phosphorus drives membrane and chlorophyll loss through H_2_O_2_ accumulation [19,41], compounded by a 10–47% reduction in light-harvesting complex components [39].

Previous studies have shown zinc deficiency to reduce ΦPSII, stomatal conductance, and transpiration in maize and other species [11,14,23,42,43,44]. The contrast with the current study seems to be linked with the timing of zinc starvation. A rice study found no decline in ΦPSII under zinc deficiency in the early vegetative stage, while the phosphorus-deficient treatment showed a decline [8]. Moreover, a longer maize study [14] detected a ΦPSII decline under zinc deficiency after two months—well beyond the 22-day period in the current study. These results, therefore, suggest zinc-driven photochemical decline is a late-onset effect not captured in the duration of the current study. Combined −Zn−P deficiency, by contrast, damaged PSII reaction centers and the oxygen-evolving complex in the early vegetative stage in rice seedlings [8], causing a decline in ΦPSII, a pattern observed in the current study as well.

**Figure 6:**
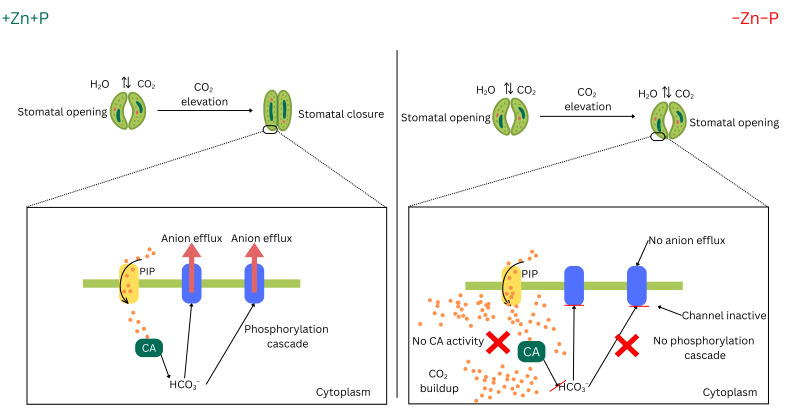
The hypothesized mechanism for high stomatal conductance in low ΦPSII and ETR where dual zinc and phosphorus deficiency inactivates the CA-regulated stomatal closure.

The combined −Zn−P treatment showed an unusually high stomatal conductance while ΦPSII and ETR remained low. We hypothesize that this decoupling reflects impaired carbonic anhydrase-mediated stomatal regulation. Phosphorus deficiency is understood to raise internal CO_2_ concentration, thereby triggering stomatal closure [45]. A mechanism involving carbonic anhydrase 4 (CA4) in Arabidopsis has been reported to drive stomatal closure in two complementary ways: catalytically, by converting accumulated CO_2_ to bicarbonate and so activating the slow anion channel-associated 1 (SLAC1) anion channel through a protein kinase cascade [46,47,48]; and directly, by binding and activating SLAC1 independently of the catalytic mechanism [46,47,48]. We hypothesize a similar mechanism in the dual deficiency where combined zinc and phosphorus deficiency could disable both mechanisms simultaneously. CA activity declines as zinc is a cofactor of the enzyme [49], while the kinase cascade needed for the catalytic route is separately impaired by low Pi [46]. The suppression of both pathways results in stomata opening despite declining ΦPSII and ETR. The hypothesized mechanism is represented in Figure 6.

### 3.2. NAR Is the Dominant Contributor to RGR Under Both Sufficiency and Deficiency

Three independent methods—PCA, LMG variance partitioning, and piecewise SEM—converged on the dominance of NAR over LAR in determining variation in whole-plant RGR across every treatment combination tested. These results are supported by previous studies on early vegetative growth, where NAR outweighed LAR in contributing towards RGR [50,51]. A wild-barley study under nutrient limitation reported the same NAR dominance across deficiency levels [52], reinforcing these observations across species and stress combinations. A study on field-grown maize under phosphorus deficiency [33] had contrasting results to the current study where leaf canopy rather than NAR was identified as the main driver of RGR. This divergence could be rooted in the difference in growth stages and differences in the sand-culture system under greenhouse conditions versus field conditions.

The piecewise SEM quantified the magnitude of NAR’s influence on RGR (β = 0.768) and showed that phosphorus availability influenced RGR predominantly through its effect on NAR (β = 0.573), which is in line with the role of phosphorus in photosynthetic electron transport and the Calvin cycle [19,53]. The SEM also identified a smaller but significant contribution from LAR (β = 0.104), consistent with the established role of phosphorus in leaf area expansion [54,55,56].

### 3.3. A Direct P-to-RGR Pathway Independent of NAR and LAR

A significant direct path from P status to RGR (β = 0.213) was identified in the piecewise SEM after accounting for mediation through NAR and LAR. This indicates that phosphorus availability influences plant growth through processes not fully captured by the leaf area-based growth components. One plausible mechanism is the specific absorption rate of phosphorus by roots (SAR[P]), defined as the amount of phosphorus absorbed per unit root dry mass per unit time. A positive relationship between SAR[P] and RGR has been documented in *Lactuca sativa*, where both NAR and SAR[P] were identified as principal determinants of RGR [57]. Therefore, we hypothesize that SAR[P] may represent a root-mediated pathway through which phosphorus availability influenced RGR independently of the measured leaf-level components.

The SEM also notably identified a significant interaction effect between phosphorus and zinc sufficiency on LAR (P × Zn, β = −0.570). This pattern was significantly attenuated in AMF-inoculated plants (P × Zn × AMF, β = 0.473, *p* < 0.05), consistent with the substantially larger AMF-associated LAR increase observed specifically in A1 (84.8 to 112 cm^2^ g^−1^; Table 4) relative to the other treatments.

The piecewise SEM explained a substantial proportion of variance across all four response variables. Marginal R^2^ ranged from 0.523 for NAR to 0.956 for RGR. The high marginal R^2^ (0.956) for RGR reflects the arithmetic relationship between RGR and its components NAR and LAR in the classical growth-analysis framework. The R^2^ for ΦPSII (0.755), on the other hand, indicates that the phosphorus, zinc, and AMF treatment structure accounts for the large majority of variance in photochemical efficiency. The comparatively lower R^2^ for NAR (0.523) reflects effects on NAR not captured by the model, possibly related to cellular respiration, a component of NAR not measured in the study.

The SEM also has some notable limitations. It failed to capture two relationships that are theoretically backed and have been empirically established by the results of this study: the positive effect of AMF on LAR under phosphorus-sufficient conditions (represented here by a non-significant path coefficient of 0.281), and the pathway from ΦPSII to NAR. The well-established coupling between ΦPSII, ETR, and photosynthetic carbon assimilation [58,59] suggests that the non-significance of the ΦPSII → NAR path may instead reflect a model structural limitation. However, the (*p* < 0.10) could reflect a trend that could possibly be captured by a longer experimental duration.

### 3.4. AMF Reduces ΦPSII in Phosphorus-Deficient Maize

In contrast to studies reporting AMF-mediated restoration of ΦPSII and ETR under phosphorus deficiency [60,61], the present study found that AMF inoculation significantly reduced ΦPSII in phosphorus-deficient treatments. The SEM’s P × AMF interaction (β = 0.408) implies a weaker phosphorus–ΦPSII association under AMF inoculation as compared to phosphorus–ΦPSII alone (β = 0.558). Moreover, the A2 (dual deficiency) treatment carried a significant negative effect on ΦPSII beyond what the main phosphorus and zinc effects alone predict (β = −0.278), and this reduction was roughly 1.8 times larger under AMF inoculation (β = −0.494). This pattern is directly corroborated by the observed treatment means, where AMF significantly reduced ΦPSII by 29.6–30.8% in A2 and A3 (*p* < 0.01; Table 2). This is consistent with AMF imposing a photochemical cost specifically under combined nutrient deficiency, plausibly related to carbon diversion toward fungal structures during active colonization. During the symbiosis, host photosynthates are drawn towards the AMF [62]. Moreover, the periarbsucular membrane formed at the site of nutrient exchange between the AMF and host has been established to be host-derived [63,64]. As AMF colonization was high (31–43%), corresponding to a metabolically active symbiosis, we hypothesize that some carbon was directed towards the AMF and periarbuscular membrane. This carbon diversion could have reduced the carbon available for maintaining the photosynthetic apparatus and could plausibly account for the observed ΦPSII/ETR suppression. However, this mechanism was not directly measured and cannot be confirmed from the present data.

The high ΦPSII and ETR observed in the deionized water control (A5), alongside low stomatal conductance and NAR, indicate a decoupling of electron transport from CO_2_ assimilation. This pattern is consistent with electron flow to alternative acceptors, notably molecular oxygen, generating reactive oxygen species (ROS) [65]. This phenomenon has been documented in maize under cold stress [66,67], heat stress [68,69], and nutrient stress [70].

### 3.5. Limitations

The study contains some methodological and analytical limitations. The experiment captured plant responses only during the first 22 days after sowing, corresponding to the early vegetative stage. Physiological and growth responses to AMF and nutrient deficiency during later vegetative development, during the reproductive stage, or at final yield cannot be inferred from these data. Moreover, plants were grown in sand culture under controlled greenhouse conditions rather than in natural agricultural soils. The medium notably lacks native microbial and AMF communities as well as the physical heterogeneity of field soils. These conditions contribute to nutrient availability and plant–AMF interactions under real growing conditions. Furthermore, only one genotype, SUWAN 5819, was evaluated. AMF responsiveness and nutrient-acquisition strategies are known to vary substantially among maize genotypes, meaning the magnitude and direction of the effects reported here may not generalize across maize germplasm. Another limitation involved not using sterilized, AMF-free inoculum carrier to isolate AMF-specific effects from those attributable to the carrier substrate itself. However, the variability in M1 treatments over M0 is unlikely to be attributed to carrier material.

One of the limitations of this study was potassium not being compensated for when KH_2_PO_4_ was removed from the −P treatments. This reduced the total solution potassium from 6 mM to 5 mM, representing a 16.7% reduction. This reduction is smaller than levels shown to affect ΦPSII and ETR in maize. A 60% reduction in solution potassium produced only a 5% decline in stomatal conductance [71]. Significant declines in net photosynthetic rate (31–35%), ΦPSII (46%), and ETR (29%) required reductions of 95% or greater in potassium [71]. A separate maize study similarly required a 97.5% reduction in solution potassium to significantly reduce net photosynthetic rate and stomatal conductance [72]. The 16.7% reduction applied here therefore falls well below these thresholds. However, the influence of potassium reduction in ΦPSII and ETR in the −P treatments cannot be fully ignored as tissue potassium was not measured.

Another limitation of the study was that zinc and phosphorus concentrations in leaf, stem, and root tissue were not directly measured. This limits the strength of mechanistic explanations linking pathways governed by internal nutrient status. Tissue nutrient analysis in future studies would allow more direct mechanistic attribution. Furthermore, relative growth rate and net assimilation rate were calculated from destructive harvests taken seven days apart (15 and 22 DAS) rather than from repeated non-destructive measurements across the growth period. The reported RGR and NAR values therefore represent average growth and assimilation rates integrated over that interval, not a continuously varying trajectory of growth and carbon gain within it.

## 4. Materials and Methods

### 4.1. Experimental Location

Maize seedlings were grown for 22 days at the Central Laboratory greenhouse, Bangkhen campus, Kasetsart University, Bangkok, Thailand (13°51′11.5″ N, 100°34′20.4″ E). Greenhouse conditions were monitored continuously throughout the experimental period. The mean daily air temperature was 31.0 °C (daily maxima 37.1–41.7 °C; minima 25.5–29.8 °C) and the mean relative humidity was 73.2%. Plants were grown under a natural photoperiod (~12.4 h). Solar light intensity was recorded in lux and converted to photosynthetic photon flux density (PPFD) using the standard sunlight conversion factor of 0.0185 μmol m^−2^ s^−1^ lux^−1^ [73], yielding a mean daytime PPFD of 253 μmol m^−2^ s^−1^ (daily means: 102–435 μmol m^−2^ s^−1^; mean daily maximum 1322 μmol m^−2^ s^−1^).

### 4.2. Plant Material and Seed Sterilization

The maize variety used was SUWAN 5819, which is a single-cross hybrid of Ki64 and Ki60, provided by the Department of Agronomy, Faculty of Agriculture Kamphaeng Saen Campus, and National Corn and Sorghum Research Center, Kasetsart University, Bangkok, Thailand. Prior to use, a modified protocol by [74] was used to surface sterilize the seeds. In brief, the seeds were soaked in 10% analytical-grade sodium hypochlorite (*v*/*v*) for 15 min and rinsed 3 times with deionized water. They were then placed on autoclaved germination paper (10 seeds per sheet) and incubated for 3 days at 25 °C and 80% relative humidity. After germination, the seedlings were transplanted into plastic pots containing 1 kg of acid-washed quartz sand. The inside of each pot was lined with a 30 μm cloth mesh to prevent sand from falling out.

### 4.3. Sand Preparation

Sand washing was performed per the modified protocol of [75]. The sand was first washed with tap water to reduce dust until the water appeared clear upon settling. Then, the sand was immersed for 2 h in 10% solution of commercial bleach (6% sodium hypochlorite) (2:1 sand:solution). After this, the sand was washed 5 times with tap water and soaked in 0.1 M HCl for 24 h. The sand was then rinsed 4 times with tap water followed by two times with reverse osmosis (RO) water, and another two times with deionized water to pH 7. After this, the sand was dried for 3 days, sieved (<1 mm), and autoclaved at 121 °C for 20 min.

### 4.4. Experimental Design and Treatments

The experiment was a randomized complete block split-plot design with six replications. Five levels of nutrient treatments were assigned to main plots, and two levels of AMF inoculation were assigned to sub-plots. Nutrient treatments were based on Hoagland solution with the following composition: 5 mM Ca(NO_3_)_2_.4H_2_O; 5 mM KNO_3_; 1 mM KH_2_PO_4_; 2 mM MgSO_4_.7H_2_O; 0.1 mM Fe-EDTA; 46 μM H_3_BO_3_; 9 μM MnCl_2_.4H_2_O; 0.32 μM CuSO_4_.5H_2_O; 0.11 μM Na_2_MoO_4_.2H_2_O; 0.75 μM ZnSO_4_.7H_2_O (Table 1). The −P treatments were modified by the removal of KH_2_PO_4_, and −Zn treatments were modified by the removal of ZnSO_4_.7H_2_O. Potassium was not compensated for in the −P treatments (see Section 3). Plants supplied with deionized water constituted the control treatment. The pH of the Hoagland solution was maintained and adjusted at 5.8–6.2 using drops of 4 M NaOH.

Nutrient treatments started at 8 days after sowing (DAS). An automated irrigation timer was used to maintain soil moisture at 70% water-holding capacity. An amount of 1 kg of dried sand was initially soaked with deionized water to full capacity to determine the weight at 100% WHC. The pots were then supplied with 65 mL daily to maintain 70% WHC, a volume determined prior to the experiment. The five nutrient treatments were: A1 (+Zn+P), A2 (−Zn−P), A3 (+Zn−P), A4 (−Zn+P), and A5 (deionized water control). AMF-inoculated sub-plots were designated M1 and non-inoculated were designated M0.

The AMF inoculant consisted of a sterilized 1:1 (*w*/*w*) mixture of sand and soil containing *Rhizophagus irregularis* at a propagule density of 25 spores g^−1^, supplied by the Soil Microbiology Research Group, Department of Agriculture, Bangkok, Thailand. An amount of 30 g of inoculant was used in the M1 treatments 7 DAS. Plants in M0 treatments did not receive any inoculant material.

### 4.5. Sampling and Measurements

Destructive sampling was conducted twice, at 15 DAS (harvest 1) and 22 DAS (harvest 2). Growth parameters (dry weights and leaf area) were measured and calculated from both harvests, while physiological measurements were taken at the second harvest. Leaf, stem, and root dry weights were obtained by drying fresh samples in a hot-air oven at 70 °C for 48 h followed by weighing on an electric balance. Chlorophyll content was measured using a Konica Minolta SPAD-502Plus Chlorophyll Meter manufactured by Konica Minolta Sensing, Inc., Osaka, Japan. Each value is a mean of three readings taken from the third fully expanded leaf from the apex at 09:00 h [76]. Stomatal conductance (gsw), ΦPSII, and ETR were measured between 11:00 and 12:00 h using LI-600 porometer/fluorometer manufactured by LI-COR Biosciences, Lincoln, NE, USA, software version 3.0.1, with each data point averaged across three readings per plant. Leaf area was estimated using the formula proposed by [77]: leaf area = leaf length × leaf width × 0.75. The correction factor used has been previously validated for SUWAN 5819 [78] and was applied uniformly across all treatment groups.

### 4.6. Derived Growth Parameters

Leaf area ratio (LAR) was calculated as described by [79]. Relative growth rate (RGR) and net assimilation rate (NAR) were calculated using the following equations [80]:

RGR = (ln W_2_ − ln W_1_)/(t_2_ − t_1_)

where W_1_ and W_2_ are dry weights of the leaf, stem, root, or total plant at times t_1_ and t_2_, respectively.

NAR = [(W_2_ − W_1_)/(t_2_ − t_1_)] × [(ln L_2_ − ln L_1_)/(L_2_ − L_1_)]

where L_1_ and L_2_ are total leaf areas at times t_1_ and t_2_, respectively.

### 4.7. AMF Colonization Assessment

Harvested root samples were heated in 10% KOH for 5–7 min, washed, and stained with trypan blue following the method of [81]. Colonization was quantified using the gridline intersection method at 45× magnification with 6 replications [82].

### 4.8. Piecewise Structural Equation Modelling

Piecewise structural equation modelling (piecewise SEM) was used to quantify the direct and indirect pathways through which phosphorus supply, zinc supply, and AMF inoculation influence whole-plant RGR. Four endogenous variables were specified: ΦPSII, NAR, LAR, and whole-plant RGR. Exogenous predictors were binary treatment indicators for phosphorus supply status (P status: 1 for A1 and A4; 0 otherwise), zinc supply status (Zn status: 1 for A1 and A3; 0 otherwise), AMF inoculation (AMF: 1 for M1; 0 for M0), and their two-way interactions P × AMF and Zn × AMF. Treatment A5 served as the reference intercept.

Each endogenous variable was modelled using a linear mixed-effects model fitted with the lmer function from the lme4 package v2.0.1 [83] with maximum likelihood estimation (REML = FALSE). Continuous endogenous variables were z-score standardized prior to model fitting. The hypothesized path structure was: P status, Zn status, AMF, P × AMF, and Zn × AMF as predictors of ΦPSII; ΦPSII and P status as predictors of NAR; ΦPSII, P status, AMF, P × AMF, and Zn × AMF as predictors of LAR; and NAR, LAR, and P status as predictors of RGR. The four component models were assembled using the psem function from the piecewiseSEM package v2.3.1 [84]. Marginal and conditional R^2^ values were calculated following [85] using the MuMIn package v1.48.19 [86].

### 4.9. Relative Importance Analysis

The LMG method [87] via the relaimpo package v2.2.7 was used to estimate the relative importance of NAR and LAR in explaining variation in RGR [88].

### 4.10. Statistical Analysis

All variables were analyzed using two-way ANOVA (*p* < 0.05) via linear mixed-effects models fitted with lmer (lme4 package v2.0.1) or lme (nlme package v3.1.169). Nutrient treatment (five levels), AMF inoculation (two levels), and their interaction were specified as fixed effects and the block (six replications) as a random effect. Normality of residuals was confirmed using the Shapiro–Wilk test (*p* > 0.05), and homogeneity of variance was assessed using Levene’s test (*p* > 0.05). Where normality assumptions were violated, log transformation was applied. For log-transformed parameters, back-transformed geometric means with geometric standard deviations (GSDs) are reported and indicated by a dagger (†) in the tables, except for Table 1 where 95% confidence intervals in parentheses are reported instead. The stem dry weight of the first destructive sampling was Box–Cox transformed as log transformation failed to restore normality; estimated marginal means are reported for this variable. Stem dry weight at harvest 2 exhibited heterogeneous variance and instead was analyzed using the lme function with a varIdent weighting structure. ΦPSII and ETR were analyzed using aligned-ranks transformation ANOVA (ART-ANOVA) via the ARTool package v0.11.2, for which raw means are reported. SS and MS are not given for ART-ANOVA as the analysis involves aligned and ranked data. Therefore, conventional sums of squares (SS) and mean squares (MS) are not directly interpretable and are typically not reported. Full ANOVA tables for physiological data (stomatal conductance, ΦPSII, and ETR) and growth parameters (dry weights and leaf area for harvests 1 and 2, LAR, and RGR for leaf, stem, root, and whole plant) have been provided in the Supplementary Materials as Table S1 and Table S2, respectively.

Post hoc pairwise comparisons of estimated marginal means were performed by Šidák adjustment [89]. Principal component analysis (PCA) was used to explore multivariate patterns among colonization, growth, and physiological variables, using the FactoMineR v2.14 and factoextra v2.0.0 packages. The number of components retained was determined by the scree plot [90] together with the Kaiser criterion [91]. All visualizations were generated using ggplot2 v4.0.3 [92]. GenAI Claude (Sonnet 4.5, Anthropic, San Francisco, CA, USA) was used for assistance in data analysis and R code generation. Each line of code was manually validated by the authors for statistical appropriateness and reproducibility. All statistical analyses were conducted in R (RStudio version 2026.04.0+526).

## 5. Conclusions

Across every analysis in this study, phosphorus status—not zinc status—was the variable that mattered for early-stage maize photochemistry and growth. Zinc deficiency alone produced no detectable effect on ΦPSII or ETR at this developmental stage, while phosphorus deficiency suppressed both consistently. Combined zinc and phosphorus deficiency produced a decoupling of stomatal aperture from photosynthetic performance, hypothesized to result from the convergent failure of CA-mediated stomatal closure. On the growth side, NAR rather than LAR accounted for most of the variance in whole-plant RGR across every treatment combination, and the piecewise SEM located a further direct phosphorus-to-RGR path independent of either. The third path is hypothesized to be root-level phosphorus absorption capacity rather than anything visible at the leaf level. Unlike in previous studies, AMF inoculation significantly reduced ΦPSII under phosphorus deficiency. These results show a system in which phosphorus availability, not zinc, sets the photosynthetic and growth ceiling in early vegetative maize in sand culture under the conditions tested. Moreover, AMF were shown to impose a cost in plant photochemistry without improved RGR in the tested conditions.

## Figures and Tables

**Table 1:** Effect of nutrient treatment and AMF inoculation on mycorrhizal root colonization in maize. † Colonization data log-transformed prior to analysis. Reported values are back-transformed geometric means with 95% confidence intervals in parentheses. Different letters indicate significant differences by Šidák adjustment (*p* < 0.05). A1 = +Zn+P; A2 = −Zn−P; A3 = +Zn−P; A4 = −Zn+P; A5 = deionized water control; M0 = without AMF; M1 = with AMF.

Treatment	AMF	Colonization (%) †
A1 (+Zn+P)	M0	4.31 (2.5–7.4) a
	M1	33.38 (19.5–57.2) b
A2 (−Zn−P)	M0	5.72 (3.3–9.8) a
	M1	31.74 (18.5–54.4) b
A3 (+Zn−P)	M0	3.53 (2.1–6.1) a
	M1	43.15 (25.2–74.0) b
A4 (−Zn+P)	M0	5.17 (3.0–8.9) a
	M1	49.59 (28.9–85.0) b
A5 (Control)	M0	2.80 (1.6–4.8) a
	M1	30.05 (17.5–51.5) b
Grand Mean		20.94
Nutrient		ns
AMF		***
Nutrient × AMF		ns

Significancecodes: *** *p* < 0.001; ns *p* ≥ 0.10.

**Table 2:** Effects of nutrient treatment and AMF inoculation on stomatal conductance, ΦPSII, and electron transport rate (ETR) in maize. † Stomatal conductance data are log-transformed prior to analysis. Log-transformed values are geometric means (×/÷GSD). ΦPSII and ETR are arithmetic means ± SD. A1 = +Zn+P; A2 = −Zn−P; A3 = +Zn−P; A4 = −Zn+P; A5 = deionized water control; M0 = without AMF; M1 = with AMF. GSD = geometric standard deviation; ΦPSII = effective quantum yield of photosystem II; ETR = electron transport rate.

Treatment	AMF	Stomatal Conductance (mol m^−2^ s^−1^) †	ΦPSII	Electron Transport Rate (μmol m^−2^ s^−1^)
		Geo (×/÷GSD)	Mean ± SD	Mean ± SD
A1 (+Zn+P)	M0	0.13 ×/÷ 1.12	0.70 ± 0.01	30.1 ± 0.5
	M1	0.17 ×/÷ 1.29	0.71 ± 0.01	30.3 ± 0.44
A2 (−Zn−P)	M0	0.26 ×/÷ 1.24	0.54 ± 0.05	23.2 ± 2.3
	M1	0.12 ×/÷ 1.08	0.38 ± 0.18	16.6 ± 7.74
A3 (+Zn−P)	M0	0.06 ×/÷ 1.07	0.52 ± 0.09	22.1 ± 4.02
	M1	0.07 ×/÷ 1.06	0.36 ± 0.13	15.2 ± 5.77
A4 (−Zn+P)	M0	0.24 ×/÷ 1.35	0.71 ± 0.01	30.5 ± 0.39
	M1	0.16 ×/÷ 1.35	0.71 ± 0.01	30.3 ± 0.55
A5 (Control)	M0	0.07 ×/÷ 1.06	0.65 ± 0.03	28.6 ± 1.02
	M1	0.05 ×/÷ 1.01	0.64 ± 0.04	28.1 ± 2.41
Grand Mean		0.18	0.59	25.50
Nutrient		*	***	***
AMF		ns	**	***
Nutrient × AMF		ns	*	**
CV/GCV (%)		65.95 †	13.52	13.34

Significance codes: *** *p* < 0.001; ** *p* < 0.01; * *p* < 0.05; ns *p* ≥ 0.10.

**Table 3:** Effects of nutrient treatment and AMF inoculation on leaf, stem, root, and whole-plant dry weights at harvest 1 (subscript 1: 15 DAS) and harvest 2 (subscript 2: 22 DAS). † Root and total dry weights for harvests 1 and 2 represent data that were log-transformed prior to analysis. Values for these variables are geometric means (×/÷GSD). § Stem dry weights for first destructive sampling represent data that were Box–Cox transformed prior to analysis. Estimated marginal means are reported for this variable (±SD). Leaf and stem dry weights are arithmetic means ± SD. LDW = leaf dry weight; SDW = stem dry weight; RDW = root dry weight; TDW = total dry weight; GSD = geometric standard deviation; DAS = days after sowing. A1 = +Zn+P; A2 = −Zn−P; A3 = +Zn−P; A4 = −Zn+P; A5 = deionized water control; M0 = without AMF; M1 = with AMF.

Treatment	AMF	LDW_2_ (g)	SDW_2_ (g)	RDW_2_ (g) †	TDW_2_ (g) †	LDW_1_ (g)	SDW_1_ (g) §	RDW_1_ (g) †	TDW_1_ (g) †
		Mean ± SD	Mean ± SD	Geo (×/÷GSD)	Geo (×/÷GSD)	Mean ± SD	Mean ± SD	Geo (×/÷GSD)	Geo (×/÷GSD)
A1 (+Zn+P)	M0	1.63 ± 0.20	0.79 ± 0.03	2.44 ×/÷ 1.54	4.91 ×/÷ 1.28	0.53 ± 0.09	0.24 ± 0.03	1.24 ×/÷ 1.62	2.05 ×/÷ 1.37
	M1	1.49 ± 0.18	0.64 ± 0.05	1.28 ×/÷ 1.38	3.45 ×/÷ 1.13	0.56 ± 0.05	0.23 ± 0.06	0.95 ×/÷ 1.94	1.81 ×/÷ 1.46
A2 (−Zn−P)	M0	0.50 ± 0.09	0.20 ± 0.03	1.00 ×/÷ 1.12	1.70 ×/÷ 1.09	0.34 ± 0.04	0.16 ± 0.03	1.21 ×/÷ 1.39	1.73 ×/÷ 1.29
	M1	0.48 ± 0.08	0.22 ± 0.09	0.63 ×/÷ 1.24	1.33 ×/÷ 1.19	0.30 ± 0.06	0.13 ± 0.04	0.98 ×/÷ 1.70	1.44 ×/÷ 1.50
A3 (+Zn−P)	M0	0.39 ± 0.14	0.23 ± 0.09	0.81 ×/÷ 1.18	1.42 ×/÷ 1.21	0.28 ± 0.11	0.12 ± 0.03	1.36 ×/÷ 1.22	1.76 ×/÷ 1.19
	M1	0.51 ± 0.16	0.19 ± 0.03	0.82 ×/÷ 1.23	1.51 ×/÷ 1.25	0.29 ± 0.04	0.14 ± 0.02	1.48 ×/÷ 1.60	1.94 ×/÷ 1.44
A4 (−Zn+P)	M0	1.66 ± 0.11	0.77 ± 0.06	1.66 ×/÷ 1.56	4.18 ×/÷ 1.17	0.53 ± 0.09	0.23 ± 0.04	1.18 ×/÷ 1.37	1.97 ×/÷ 1.21
	M1	1.53 ± 0.14	0.68 ± 0.09	1.67 ×/÷ 1.41	3.91 ×/÷ 1.19	0.50 ± 0.05	0.26 ± 0.19	0.83 ×/÷ 1.21	1.64 ×/÷ 1.15
A5 (Control)	M0	0.23 ± 0.06	0.09 ± 0.02	0.75 ×/÷ 1.36	1.09 ×/÷ 1.22	0.17 ± 0.05	0.07 ± 0.01	0.63 ×/÷ 1.42	0.88 ×/÷ 1.28
	M1	0.19 ± 0.08	0.10 ± 0.01	0.58 ×/÷ 1.38	0.87 ×/÷ 1.25	0.14 ± 0.06	0.08 ± 0.01	0.64 ×/÷ 1.77	0.89 ×/÷ 1.58
Grand Mean		0.86	0.39	1.16	2.44	0.37	0.18	1.13	1.68
Nutrient		***	***	***	***	***	***	*	**
AMF		ns	ns	**	***	ns	ns	ns	ns
Nutrient × AMF		*	***	*	*	ns	ns	ns	ns
CV (%)		10.70	13.15	31.20 †	16.82 †	18.80	37.90	38.50 †	27.90 †

Significance codes: *** *p* < 0.001; ** *p* < 0.01; * *p* < 0.05; ns *p* ≥ 0.10.

**Table 4:** Effects of nutrient treatment and AMF inoculation on leaf area, leaf area ratio (LAR), leaf, stem, root, and whole-plant relative growth rate (RGR), and net assimilation rate (NAR). † Leaf area data at harvests 1 and 2 represent data that were log-transformed prior to analysis. Values for these variables are geometric means (×/÷GSD). LAR, RGRs, and NAR are arithmetic means ± SD. LA_1_ = leaf area at harvest 1; LA_2_ = leaf area at harvest 2; LAR = leaf area ratio; RGR = relative growth rate; NAR = net assimilation rate; GSD = geometric standard deviation. A1 = +Zn+P; A2 = −Zn−P; A3 = +Zn−P; A4 = −Zn+P; A5 = deionized water control; M0 = without AMF; M1 = with AMF.

Treatment	AMF	LA_2_ (cm^2^) †	LA_1_ (cm^2^) †	LAR (cm^2^ g^−1^)	Leaf RGR (g g^−1^ d^−1^)	Stem RGR (g g^−1^ d^−1^)	Root RGR (g g^−1^ d^−1^)	Total RGR (g g^−1^ d^−1^)	NAR (g cm^−2^ d^−1^)
		Geo (×/÷GSD)	Geo (×/÷GSD)	Mean ± SD	Mean ± SD	Mean ± SD	Mean ± SD	Mean ± SD	Mean ± SD
A1 (+Zn+P)	M0	404.3 ×/÷ 1.06	130.6 ×/÷ 1.09	84.8 ± 19.3	0.16 ± 0.03	0.17 ± 0.02	0.10 ± 0.06	0.12 ± 0.04	0.0017 ± 0.0006
	M1	370.6 ×/÷ 1.27	131.5 ×/÷ 1.09	112 ± 31.5	0.14 ± 0.02	0.14 ± 0.04	0.04 ± 0.09	0.092 ± 0.05	0.0010 ± 0.0005
A2 (−Zn−P)	M0	97.6 ×/÷ 1.07	62.7 ×/÷ 1.09	57.5 ± 5.08	0.05 ± 0.04	0.03 ± 0.05	−0.03 ± 0.06	−0.002 ± 0.04	−0.00013 ± 0.0009
	M1	91.1 ×/÷ 1.13	58.1 ×/÷ 1.10	69.0 ± 8.61	0.07 ± 0.03	0.06 ± 0.07	−0.064 ± 0.07	−0.012 ± 0.05	−0.00041 ± 0.001
A3 (+Zn−P)	M0	104.5 ×/÷ 1.35	61.9 ×/÷ 1.12	75.1 ± 17.4	0.05 ± 0.09	0.08 ± 0.07	−0.07 ± 0.04	−0.031 ± 0.05	−0.00068 ± 0.0009
	M1	95.5 ×/÷ 1.20	59.4 ×/÷ 1.09	64.6 ± 14.4	0.07 ± 0.05	0.04 ± 0.03	−0.084 ± 0.04	−0.037 ± 0.04	−0.00092 ± 0.001
A4 (−Zn+P)	M0	428.8 ×/÷ 1.04	141.6 ×/÷ 1.07	103 ± 14.6	0.16 ± 0.02	0.17 ± 0.03	0.05 ± 0.06	0.11 ± 0.02	0.00012 ± 0.0003
	M1	433.5 ×/÷ 1.06	127.3 ×/÷ 1.09	113 ± 23.1	0.16 ± 0.01	0.13 ± 0.08	0.099 ± 0.06	0.12 ± 0.02	0.0013 ± 0.0004
A5 (Control)	M0	69.9 ×/÷ 1.28	51.6 ×/÷ 1.05	68.8 ± 24.9	0.05 ± 0.06	0.04 ± 0.05	0.03 ± 0.03	0.031 ± 0.03	0.00049 ± 0.0005
	M1	70.9 ×/÷ 1.43	54.9 ×/÷ 1.08	83.4 ± 19.0	0.05 ± 0.10	0.03 ± 0.02	−0.017 ± 0.07	−0.002 ± 0.06	−0.00017 ± 0.0009
Grand Mean		216.75	87.94	83.1	0.097	0.089	0.011	0.039	0.00029
Nutrient		***	***	***	***	***	***	***	***
AMF		ns	ns	*	ns	ns	ns	ns	ns
Nutrient × AMF		ns	ns	ns	ns	ns	ns	ns	ns
CV (%)		20.05 †	8.81 †	23.0	5.09	5.02	6.17	3.78	0.072

Significance codes: *** *p* < 0.001; * *p* < 0.05; ns *p* ≥ 0.10.

**Table 5:** Relative importance (%) of net assimilation rate (NAR) and leaf area ratio (LAR) in explaining variation in whole-plant relative growth rate (RGR), estimated by the Lindeman Merenda Gold (LMG) variance partitioning method. NAR = net assimilation rate (g cm^−2^ d^−1^); LAR = leaf area ratio (cm^2^ g^−1^). A1 = +Zn+P; A2 = −Zn−P; A3 = +Zn−P; A4 = −Zn+P; A5 = deionized water control; M0 = without AMF; M1 = with AMF.

Treatment	AMF	NAR (%)	LAR (%)
A1 (+Zn+P)	M0	80.9	19.1
	M1	78.5	21.5
A2 (−Zn−P)	M0	89.0	11.0
	M1	83.9	16.1
A3 (+Zn−P)	M0	95.7	4.3
	M1	91.8	8.2
A4 (−Zn+P)	M0	75.1	24.9
	M1	62.9	37.1
A5 (Control)	M0	95.5	4.5
	M1	99.2	0.8

**Table 6:** Standardized path coefficients from the piecewise structural equation model (SEM) relating P supply, Zn supply, dual deficiency (A2), and AMF inoculation to ΦPSII, NAR, LAR, and whole-plant RGR. Models fitted with lmer (REML = FALSE) and random effects (1|Block/Nutrient). β = standardized path coefficient. ΦPSII = quantum efficiency of photosystem II, NAR = net assimilation rate, LAR = leaf area ratio, RGR = relative growth rate.

Predictor	Response	β	Significance
P status	ΦPSII	0.558	***
Zn status	ΦPSII	−0.088	ns
P × Zn	ΦPSII	0.052	ns
AMF	ΦPSII	−0.017	ns
P × AMF	ΦPSII	0.408	*
Zn × AMF	ΦPSII	−0.017	ns
P × Zn × AMF	ΦPSII	0.028	ns
A2 (−Zn−P)	ΦPSII	−0.278	*
A2 × AMF	ΦPSII	−0.494	*
ΦPSII	NAR	0.203	.
P status	NAR	0.573	***
ΦPSII	LAR	−0.080	ns
P status	LAR	0.915	***
Zn status	LAR	0.327	.
P × Zn	LAR	−0.570	*
AMF	LAR	0.281	ns
P × AMF	LAR	0.000	ns
Zn × AMF	LAR	−0.343	ns
P × Zn × AMF	LAR	0.473	*
A2 (−Zn−P)	LAR	−0.197	ns
A2 × AMF	LAR	−0.097	ns
NAR	RGR	0.768	***
LAR	RGR	0.104	**
P status	RGR	0.213	***

R^2^ (marginal/conditional): ΦPSII = 0.755/0.761; NAR = 0.523/0.596; LAR = 0.544/0.544; RGR = 0.956/0.956. Marginal R^2^ reflects fixed effects only; conditional R^2^ adds the random effects (1|Block/Nutrient). Significance codes: *** *p* < 0.001; ** *p* < 0.01; * *p* < 0.05; *p* < 0.10; ns *p* ≥ 0.10.

## Data Availability

The original contributions presented in this study are included in the article/Supplementary Material. Further inquiries can be directed to the corresponding author.

## Supplementary Materials

The following supporting information can be downloaded at https://www.mdpi.com/article/10.3390/plants15142215/s1: Table S1: Analysis of variance of physiological parameters for zinc and phosphorus deficiency with and without mycorrhiza; Table S2: Analysis of variance of biomass and leaf area parameters for harvests 1 and 2, and RGR for leaf, stem, root, and whole plant for zinc and phosphorus deficiency with and without mycorrhiza.

## Abbreviations

The following abbreviations are used in this manuscript:

AMF	Arbuscular Mycorrhiza Fungi
PSII	Photosystem II
ETR	Electron Transport Rate
RGR	Relative Growth Rate
NAR	Net Assimilation Rate
LAR	Leaf Area Ratio
CA	Carbonic Anhydrase
DAS	Days After Sowing
SEM	Structural Equation Model
SAR[P]	Specific Absorption Rate of Phosphorus
SLAC	Slow Anion Channel-Associated
ATP	Adenosine Triphosphate
RCBD	Randomized Complete Block Design
ROS	Reactive Oxygen Species
RO	Reverse Osmosis
DI	Deionized
PCA	Principal Component Analysis
LMG	Lindeman–Merenda–Gold
Pi	Inorganic Phosphate
ΦPSII	Quantum Efficiency of Photosystem II
gsw	Stomatal Conductance
ART-ANOVA	Aligned-Ranks Transformation Analysis of Variance
PPFD	Photosynthetic Photon Flux Density
