# Supplementary material for: Arbuscular Mycorrhiza Fungi Reduce Photosystem II Efficiency in Phosphorus-Deficient Maize Without Promoting Growth
Source: Plants (Basel). 2026 Jul 21;15(14):2215. doi: 10.3390/plants15142215 (PMC13416473; doi:10.3390/plants15142215)
Supplement: Supplementary file 1 [file plants-15-02215-s001.zip › plants-4436957-supplementary.pdf]

Supplementary Materials

Table S1: Analysis of variance of physiological parameters for zinc and phosphorus deficiency with and without mycorrhiza

Variable	Source of Variation	df	SS	MS	F-value	p-value	Significant codes
Stomatal Conductance (gsw) (ln) †	Nutrient	4	5.1798	1.2950	4.309	0.007	**
	AMF	1	0.6239	0.6239	2.076	0.160	ns
	Nutrient × AMF	4	1.7499	0.4375	1.456	0.240	ns
ΦPSII (ART) ‡	Nutrient	4	—	—	74.093	< 0.001	***
	AMF	1	—	—	13.275	0.001	**
	Nutrient × AMF	4	—	—	4.079	0.011	*
ETR (ART) ‡	Nutrient	4	—	—	40.297	< 0.001	***
	AMF	1	—	—	14.062	< 0.001	***
	Nutrient × AMF	4	—	—	4.256	0.009	**

AMF: Arbuscular Mycorrhiza Fungi; df: degree of freedom; SS: Sum Square; MS: Mean Square. Significance codes: *** p < 0.001; ** p < 0.01; * p < 0.05; ns: not significant. ‡ ΦPSII and ETR were non-normally distributed and analysed by the Aligned Rank Transform (ART) ANOVA. † Stomatal conductance (gsw) was log-transformed prior to analysis.

Table S2: Analysis of variance of biomass and leaf area parameters for harvests 1 and 2, and RGR for leaf, stem, root, and whole plant for zinc and phosphorus deficiency with and without mycorrhiza

Variable	Source of Variation	df	SS	MS	F-value	p-value	Significant codes
AMF Colonisation (ln) †	Nutrient	4	1.579	0.395	1.044	0.410	ns
	AMF	1	71.235	71.235	188.322	< 0.001	***
	Nutrient × AMF	4	1.148	0.287	0.759	0.562	ns
Leaf Dry Weight$_2$	Nutrient	4	7.122	1.781	209.170	< 0.001	***
	AMF	1	0.023	0.023	2.750	0.110	ns
	Nutrient × AMF	4	0.127	0.032	3.742	0.016	*
Stem Dry Weight$_2$ †	Nutrient	4	—	—	479.448	< 0.001	***
	AMF	1	—	—	38.460	< 0.001	***
	Nutrient × AMF	4	—	—	10.882	< 0.001	***
Root Dry Weight$_2$ (ln) †	Nutrient	4	10.118	2.529	27.228	< 0.001	***
	AMF	1	1.113	1.113	11.976	0.001	**
	Nutrient × AMF	4	1.026	0.256	2.761	0.038	*
Total Dry Weight$_2$ (ln) †	Nutrient	4	14.008	3.502	125.430	< 0.001	***
	AMF	1	0.412	0.412	14.759	< 0.001	***

	Nutrient × AMF	4	0.312	0.078	2.792	0.048	*
Leaf Area$_2$ (ln) †	Nutrient	4	29.556	7.389	205.606	< 0.001	***
	AMF	1	0.029	0.029	0.818	0.374	ns
	Nutrient × AMF	4	0.033	0.008	0.228	0.920	ns
Leaf Area Ratio	Nutrient	4	17,606.9	4,401.7	11.891	< 0.001	***
	AMF	1	1,691.7	1,691.7	4.570	0.037	*
	Nutrient × AMF	4	2,294.2	573.6	1.549	0.202	ns
Leaf Dry Weight$_1$	Nutrient	4	1.296	0.324	68.917	< 0.001	***
	AMF	1	0.002	0.002	0.463	0.499	ns
	Nutrient × AMF	4	0.011	0.003	0.578	0.680	ns
Stem Dry Weight$_1$ (BC) §	Nutrient	4	7.394	1.848	60.720	< 0.001	***
	AMF	1	0.024	0.024	0.783	0.385	ns
	Nutrient × AMF	4	0.155	0.039	1.269	0.309	ns
Root Dry Weight$_1$ (ln) †	Nutrient	4	2.091	0.523	4.053	0.011	*
	AMF	1	0.301	0.301	2.333	0.139	ns
	Nutrient × AMF	4	0.436	0.109	0.846	0.510	ns
Total Dry Weight$_1$ (ln) †	Nutrient	4	2.911	0.728	10.294	< 0.001	***
	AMF	1	0.085	0.085	1.203	0.283	ns
	Nutrient × AMF	4	0.190	0.048	0.673	0.617	ns
Leaf Area$_1$ (ln) †	Nutrient	4	10.008	2.502	323.607	< 0.001	***
	AMF	1	0.015	0.015	1.885	0.176	ns
	Nutrient × AMF	4	0.054	0.013	1.738	0.156	ns
RGR_l	Nutrient	4	0.123	0.031	11.892	< 0.001	***
	AMF	1	0.000	0.000	0.081	0.778	ns
	Nutrient × AMF	4	0.003	0.001	0.310	0.869	ns
RGR_s	Nutrient	4	0.159	0.040	15.760	< 0.001	***
	AMF	1	0.005	0.005	1.982	0.172	ns
	Nutrient × AMF	4	0.011	0.003	1.045	0.404	ns
RGR_r	Nutrient	4	0.222	0.056	14.587	< 0.001	***
	AMF	1	0.005	0.005	1.374	0.247	ns
	Nutrient × AMF	4	0.021	0.005	1.402	0.247	ns
RGR_t	Nutrient	4	0.171	0.043	30.003	< 0.001	***
	AMF	1	0.003	0.003	1.755	0.197	ns
	Nutrient × AMF	4	0.005	0.001	0.906	0.476	ns
NAR	Nutrient	4	3.3e-05	8.3e-06	16.002	< 0.001	***
	AMF	1	1.9e-06	1.9e-06	3.706	0.066	.
	Nutrient × AMF	4	1.4e-06	3.4e-07	0.665	0.622	ns

AMF: Arbuscular Mycorrhiza Fungi; RGR_l= relative growth rate leaf, RGR_s= relative growth rate stem, RGR_r= relative growth rate root, RGR_t= relative growth rate whole plant; df: degree of freedom; SS: Sum Square; MS: Mean Square. Significance codes: *** $p < 0.001$; ** $p < 0.01$; * $p < 0.05$; . $p < 0.10$; ns: not significant. SS and MS are not available for models fitted with heterogeneous variance structure (lme with varIdent). Stem Dry Weight and Specific Leaf Area: fitted with lme/varIdent. (ln) † represent variables that have been log-transformed prior to analysis. § Stem Dry $Weight_1$ (BC) was Box-Cox transformed (λ = -0.37) prior to analysis.
